# Supplementary material for: Explainable deep learning for tumor dynamic modeling and overall survival prediction using Neural-ODE
Source: NPJ Syst Biol Appl. 2023 Nov 18;9:58. doi: 10.1038/s41540-023-00317-1 (PMC10657412; doi:10.1038/s41540-023-00317-1)
Supplement: Supplementary file 1 — Supplementary Materials [file 41540_2023_317_MOESM1_ESM.pdf]

Supplementary Material:
"Explainable Deep Learning for Tumor Dynamic Modeling and Overall Survival Prediction using Neural-ODE"

Mark Laurie, James Lu

Supplementary Note 1

The model code available at:

- Github: https://github.com/jameslu01/TDNODE
- Zenodo: DOI 10.5281/zenodo.8436518

Hyperparameter	Value
Batch Size	8
Learning Rate	5.0e-5
Optimization	ADAM
L2 Weight Decay	1.0e-3
Padding	Left-padding w/ first measurement
Epochs	150
Observation Window (weeks)	32
NODE Tolerance	1.0e-4
Tumor State Dimensionality (c)	4
Parameter Encoding dimensionality (v)	2

Supplementary Table 1: Select hyperparameter configurations for TDNODE. *We selected and tuned hyperparameters based on empirically derived tumor dynamic predictivity and the OS c-index obtained from the XGBoost-ML model that was fitted with TDNODE-generated kinetic rate parameters. Seeking a model that could converge quickly, we also tuned hyperparameters, such as batch size, L2 Weight Decay, and NODE tolerance, considering the required optimization runtime.*

Covariate	Description
CRP	C-reactive protein (mg/L)
BECOG	Baseline Eastern Cooperative Oncology Group (ECOG) Performance Status
LDH	Lactate Dehydrogenase (U/L)
NEU	Neutrophil Count (10^9/L)
METSITES	Number of Metastatic Sites at Enrollment
TPRO	Total Protein (g/L)
YSD	Number of Years Since Diagnosis (yr)
LIVER	Number of Liver Metastatic Sites at Enrollment
BNLR	Baseline Neutrophil to Lymphocyte Ratio
ALBU	Albumin (g/L)
HGB	Hemoglobin (g/L)

Supplementary Table 2: the set of baseline covariates used in the IMPower150 dataset. *The values of the baseline covariate were used within the XGBoost-ML models in addition to TDNODE-generated kinetic rate parameters.*

Parameter	Description	Value
eta	Step size shrinkage	0.0116
max_depth	Maximum tree depth	5
min_child_weight	Child tree minimum required instance weight sum	0.0211
reg_alpha	L1 regularization term	0.0014
reg_lambda	L2 regularization term	3.415
subsample	Subsample ration to training instances	0.849
objective	Task specification	survival:cox

Supplementary Table 3**: **The set of hyperparameters used in the XGBoost-ML models in predicting patients' OS.

Treatment Arm	Number of Measurements $t > w_i$	RMSE (median±MAD)	R^2 Score (median±MAD)
Arm 1: Atezo.+Carb.+Pac.	2,112	9.56±0.22	0.95±0.003
Arm 2: Atezo.+Carb.+Pac.+Bev.	2,453	8.72±0.18	0.94±0.003
Arm 3: Carb.+Pac.+Bev.	1,882	7.51±0.16	0.96±0.002
**All Treatment arms**	**6,447**	**8.66±0.12**	**0.95±0.002**

Supplementary Table 4: Tumor dynamic predictivity of TDNODE measured via RMSE and R^2. *For each patient, we let $w_i = 32$ weeks and only evaluate measurements collected beyond w_i. As displayed in the test set (**Table 1**), RMSE and R^2 scores are calculated using only the discrete set of predictions with corresponding observed SLD measurements. Variance was measured via median absolute deviation (MAD).*

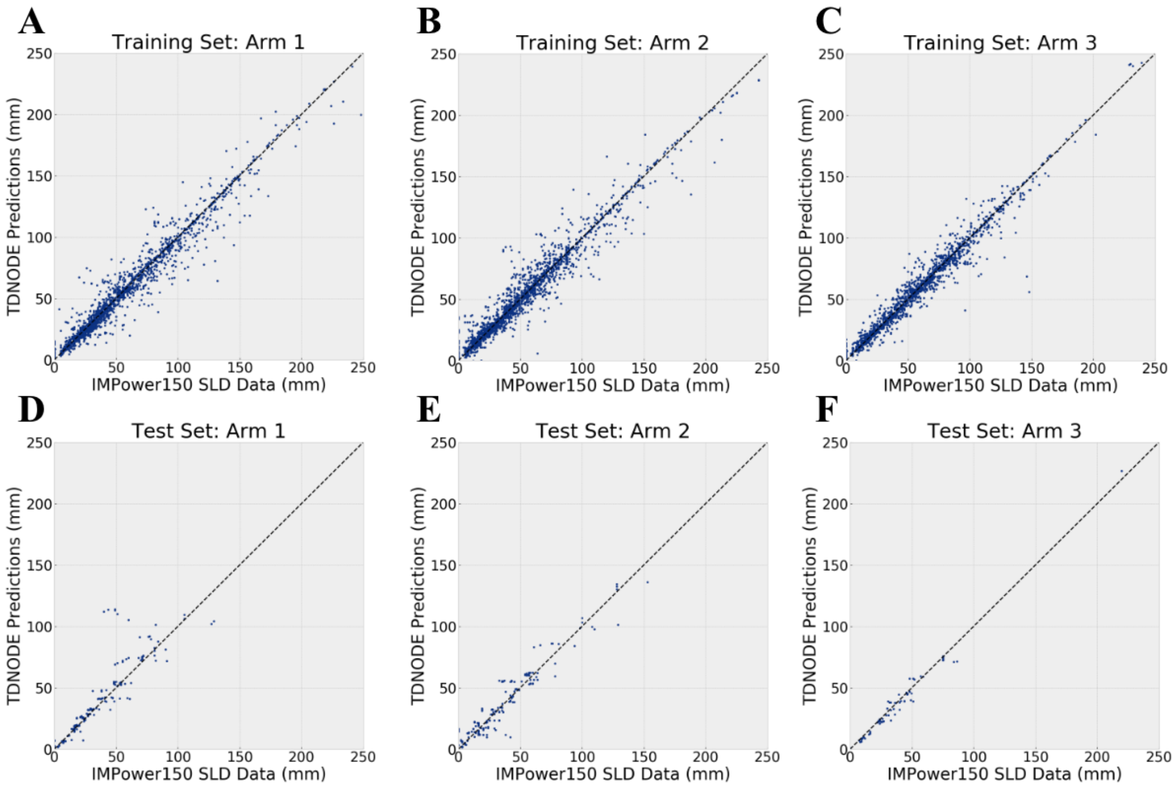

Supplementary Figure 1: TDNODE enables unbiased tumor size predictions with respect to observed tumor size. ***A-C**, Training set comparisons using all observed SLD data with respect to treatment arms 1, 2 and 3, respectively.* ***D-F**, Test set comparisons using only unseen SLD data with a 32-weeks observation window. Dashed lines denote the line of unity for each plot.*

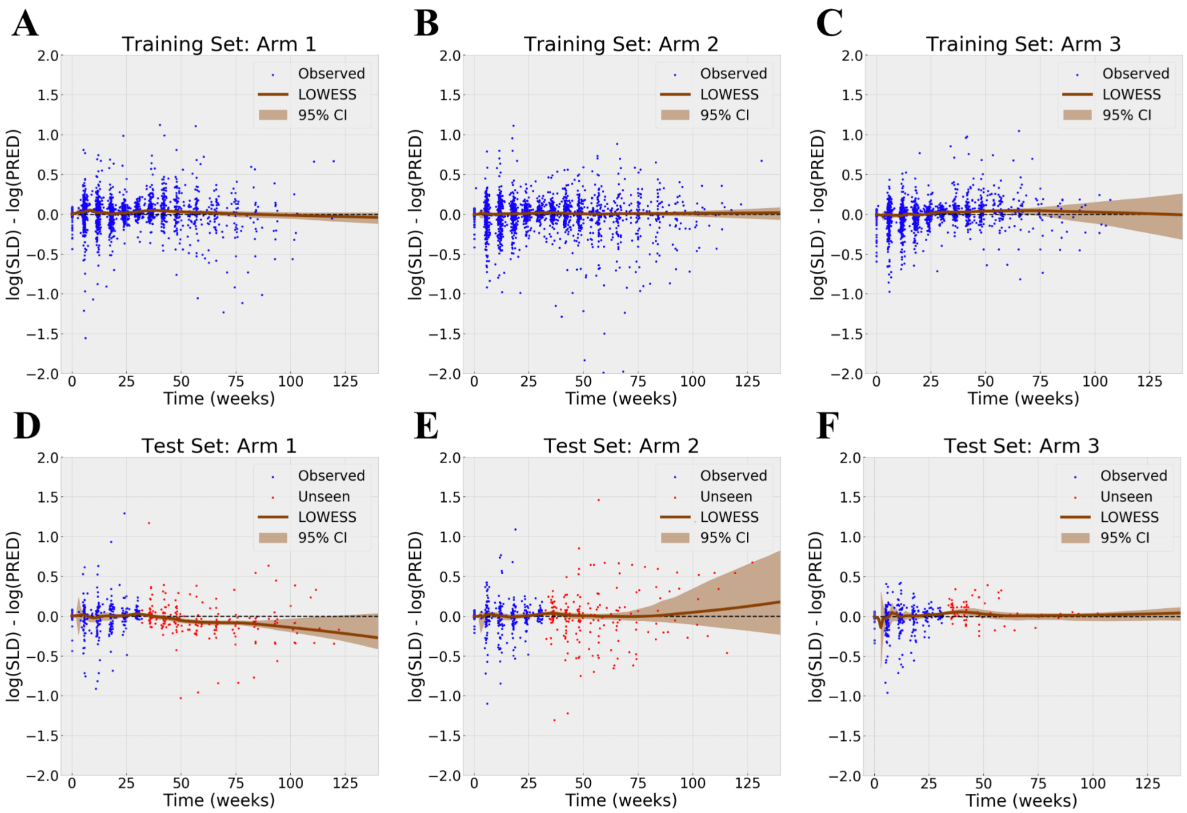

Supplementary Figure 2: TDNODE enables unbiased predictions of tumor dynamics with respect to observation time. A-C. *Training set residuals using all observed SLD data with respect to time for treatment arms 1, 2 and 3, respectively.* ***D-F,*** *Test set residuals using all observed SLD data with respect to time for treatment arms 1, 2 and 3, respectively.*

**Observation Window** (weeks)	**Number of Predictions for** $t > w_i$	**RMSE** (median$\pm$MAD)	**R^2 Score** (median$\pm$MAD)
16	928	13.48$\pm$0.47	0.82$\pm$0.02
20	768	12.22$\pm$0.51	0.83$\pm$0.02
24	682	10.52$\pm$0.51	0.87$\pm$0.02
28	616	10.89$\pm$0.59	0.86$\pm$0.02
32	501	9.69$\pm$1.02	0.88$\pm$0.03

Supplementary Table 5: Tumor dynamic predictive performance of TDNODE upon variation of w_i, measured via RMSE and R^2. *Here we observe that TDNODE's predictive performance increases as the observations window for each patient is increased, as displayed by increasing RMSE and decreasing R^2 values. Variance is captured using median absolute deviation (MAD).*

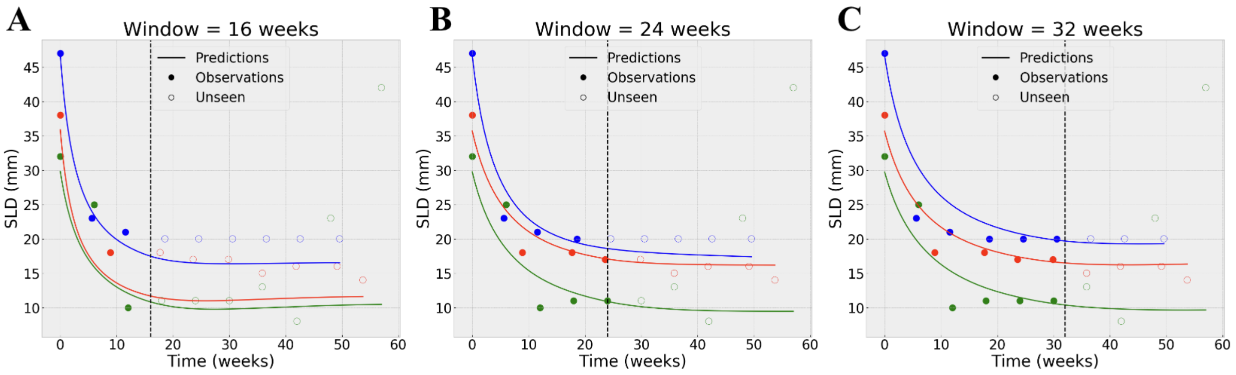

Supplementary Figure 3: Illustration of tumor dynamic predictions using TDNODE with varying observation windows for selected test patients, showing increasingly more accurate predictions as the observation window is increased. ***A-C****, TDNODE tumor dynamic predictions for select test patients when* w_i *= 16 weeks (****A****),* w_i *= 24 weeks (****B****),* w_i *= 32 weeks (****C****).*

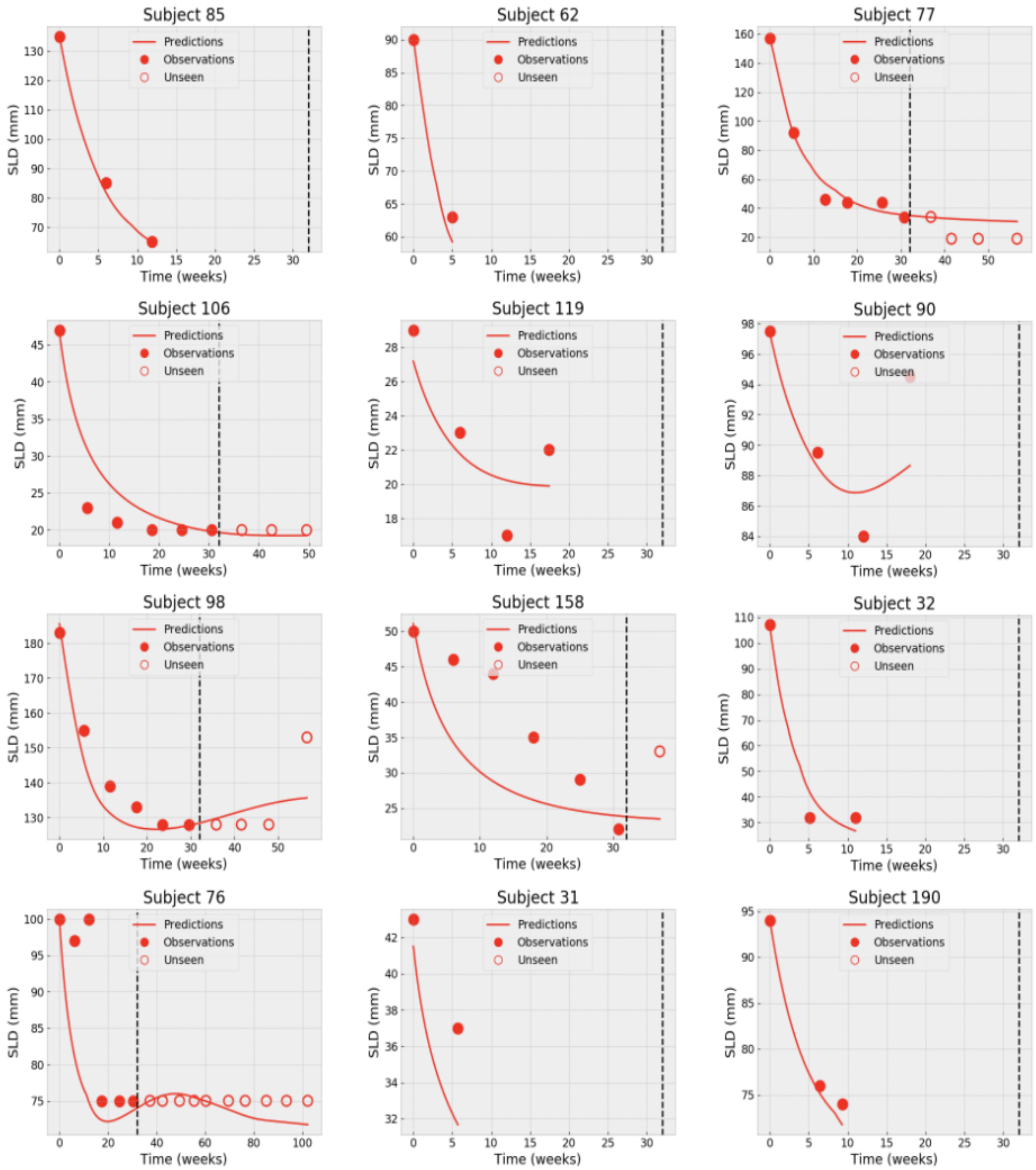

Supplementary Figure 4: TDNODE individual prediction curves using a randomly selected sample of patients from the test set. *Here we observe how TDNODE performs on a set of patients with varying observed tumor dynamic profiles (e.g. remission, bimodal, failure) and quantity of observations in the test set with a 32-week observation window.*

**Input features**	**C-index evaluated via 5-fold cross-validation**<br><br>(median±STD)	**C-index evaluated on test set**
TDNODE metrics	0.84±0.02	0.82
TDNODE metrics + 11 baseline covariates	0.86±0.02	0.84
TDNODE Principal Components	0.84±0.02	0.82
TDNODE Principal Components + 11 baseline covariates	0.85±0.02	0.84
TDNODE Parameter Encoding 1st Principal Component	0.83±0.02	0.81
TDNODE Parameter Encoding 1st Principal Component + 11 baseline covariates	0.84±0.02	0.83

Supplementary Table 6: OS performance of 6 XGBoost-ML models trained with different sets of inputs. *Here we fitted 6 XGBoost-ML models with different sets of input data. We see that including baseline covariates do not significantly impact productivity of the OS. Using only the 1st principal component derived from the kinetic rate parameter distribution, we see that OS predictivity is comparable to that of using the original kinetic rate metrics.*

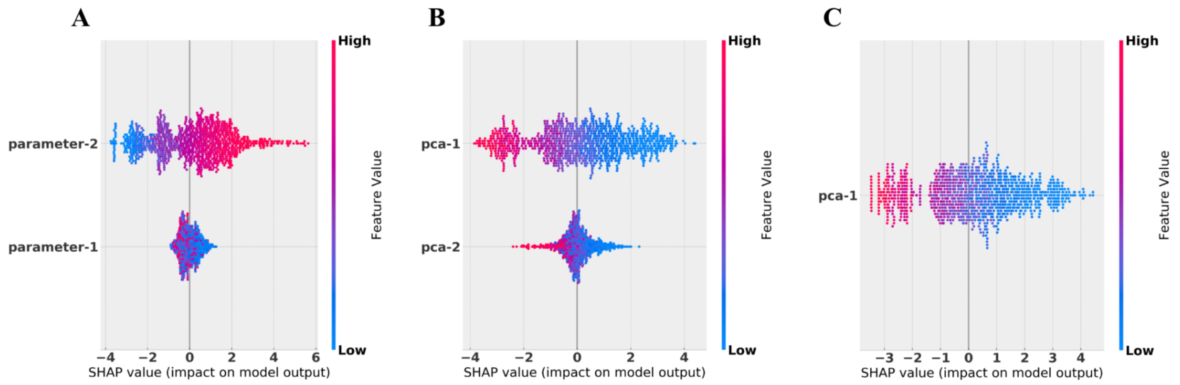

***Supplementary Figure 5: SHAP summary plot of XGBoost models utilizing TDNODE-derived metrics without baseline covariates**. **A**) SHAP summary plot of XGBoost model using TDNODE-generated parameter encodings. **B)** SHAP summary plot of XGBoost model using the two most informative principal components of the encoding distribution. **C**) SHAP summary plot of XGBoost model using just the first principal component derived from the encoding distribution.*

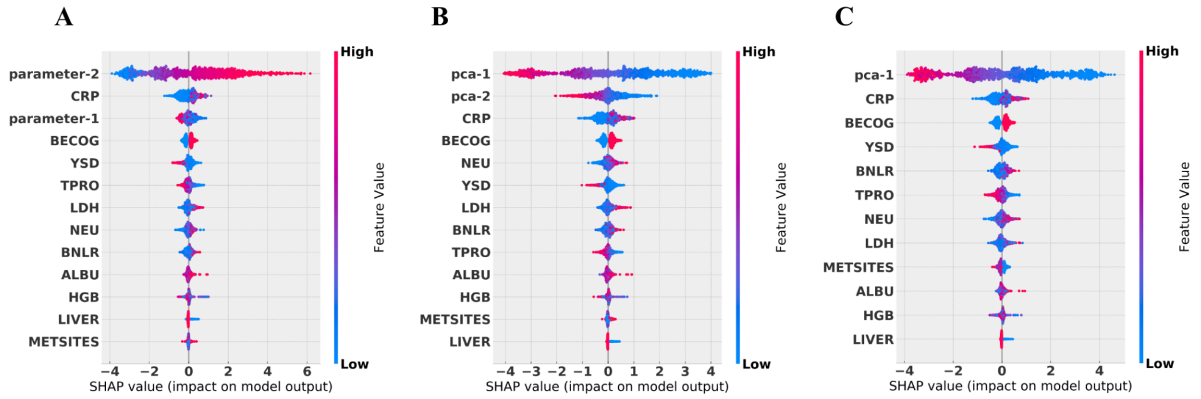

***Supplementary Figure 6: SHAP summary plot of XGBoost models utilizing TDNODE metrics with baseline covariates**. **A) S**HAP summary plot of XGBoost model using TDNODE-generated parameter encodings. **B)** SHAP summary plot of XGBoost model using the two most informative principal components of the encoding distribution. **C**) SHAP summary plot of XGBoost model using just the first principal component derived from the encoding distribution.*

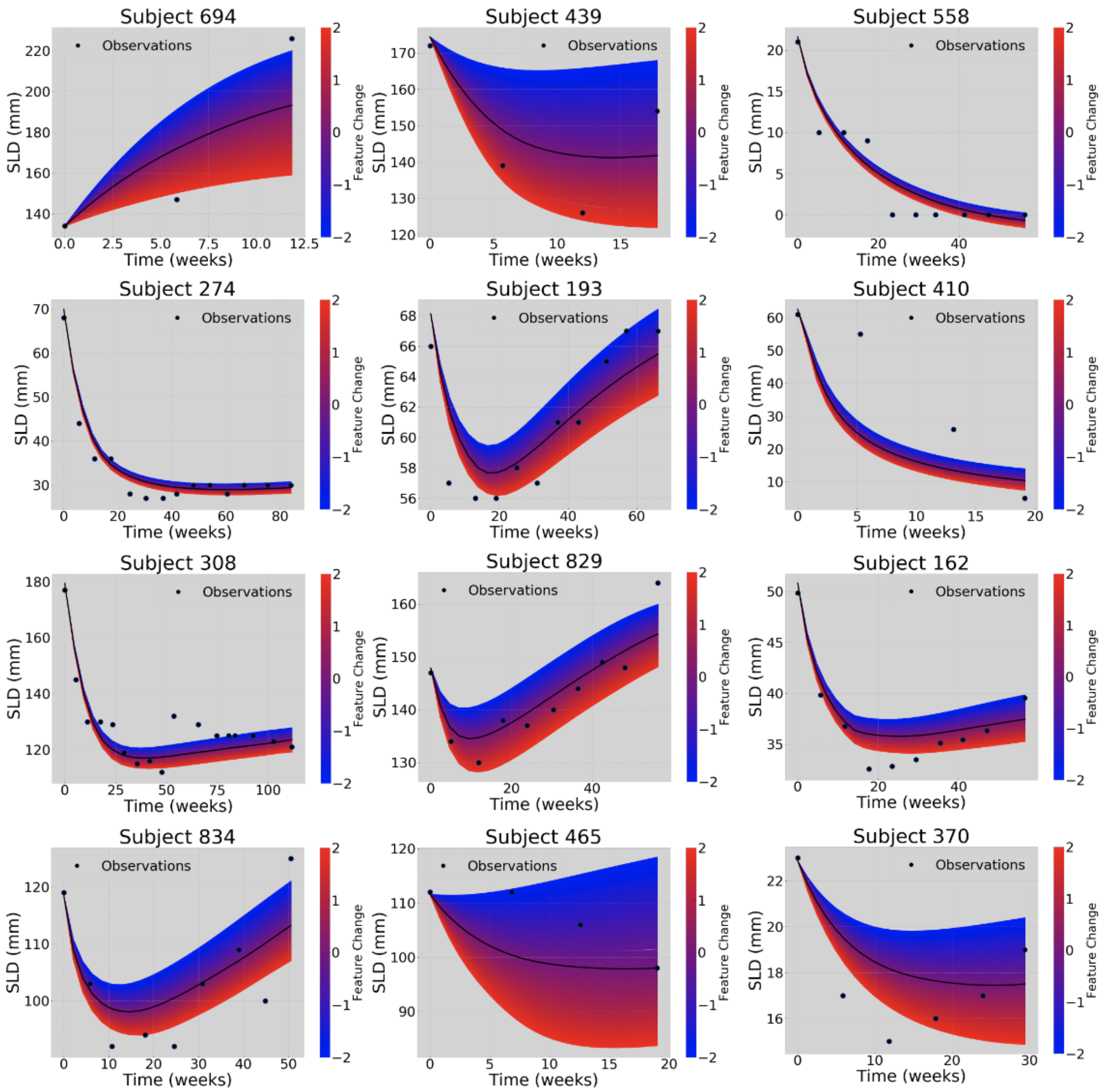

Supplementary Figure 7: Additional feature dependence plots with perturbations of the first principal component calculated from the encoding distribution. *Color bar values correspond to the magnitude of change along the direction of the first principal component from that predicted by TDNODE, which is projected into the 2-dimensional parameter encoder space. Here we observe that increases in the value of the first principal component results in a decrease in the predicted tumor size. This finding corresponds with that derived from SHAP analysis of this principal component when used in XGBoost to predict OS, where increases in this principal component result in a negative change in corresponding patients' hazard rate.*

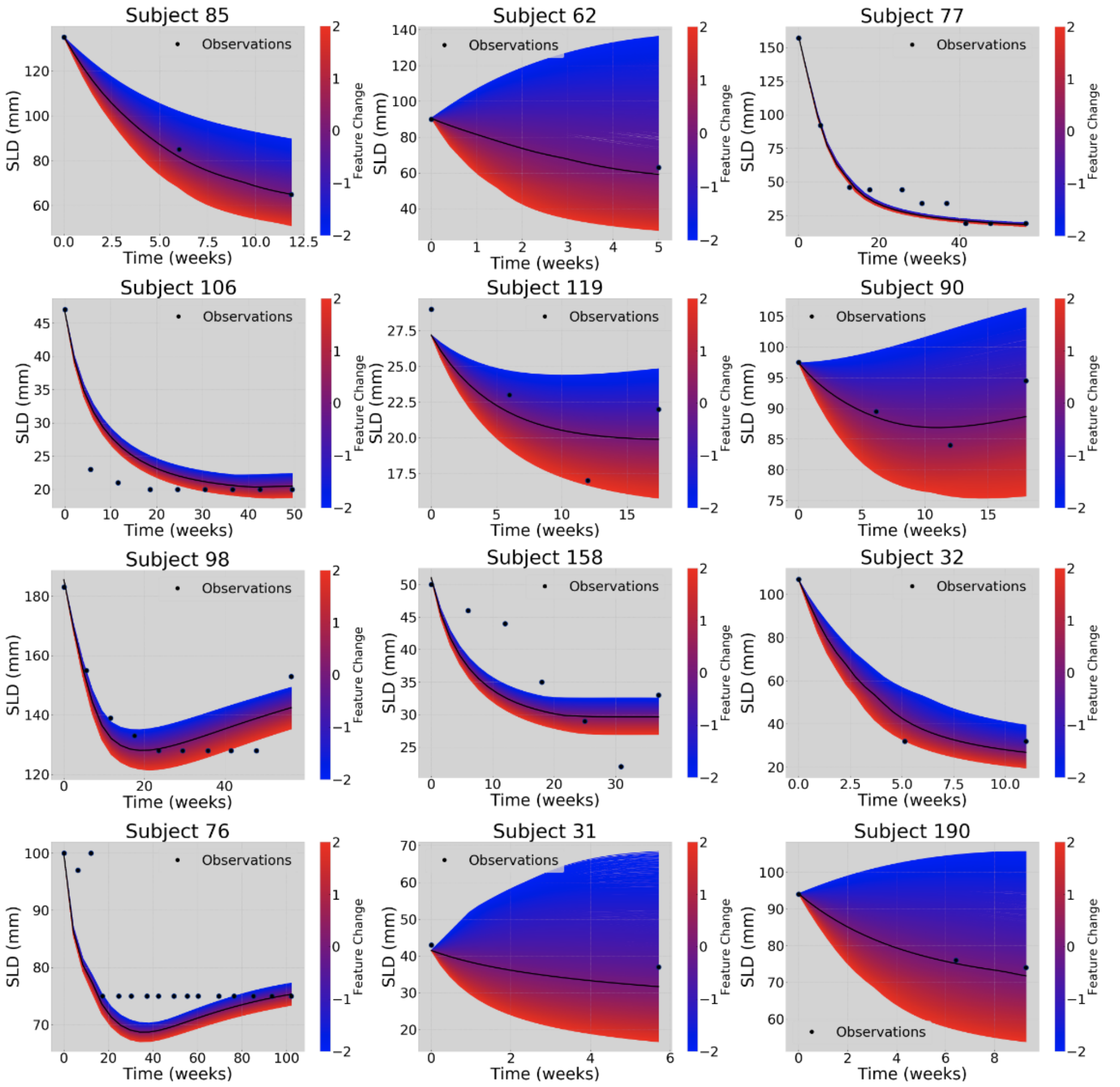

Supplementary Figure 8: Additional feature dependence plots with systematic perturbations of kinetic parameters along the first principal component from their nominal values. *Color bar values correspond to the magnitude of change along the direction of the first principal component from that predicted by TDNODE, Changes in the 1st principal axis are projected into the 2-dimensional parameter encoder space. We use the same subjects as in* ***Supplementary Figure 4.*** *Like in the training set, we observe that increases in the value of the first principal component results in a decrease in the predicted tumor size. This finding corresponds with that derived from SHAP analysis of this principal component when used in XGBoost to predict OS, where increases in this principal component result in a net negative change in hazard rate.*
